# Polyvagal Theory: A Science of Safety

**DOI:** 10.3389/fnint.2022.871227

**Published:** 2022-05-10

**Authors:** Stephen W. Porges

**Affiliations:** ^1^Traumatic Stress Research Consortium, Kinsey Institute, Indiana University, Bloomington, IN, United States; ^2^Department of Psychiatry, University of North Carolina at Chapel Hill, Chapel Hill, NC, United States

**Keywords:** polyvagal theory, autonomic nervous system, ventral vagal complex, social engagement system, threat reactions, feelings of safety, neuroception

## Abstract

Contemporary strategies for health and wellbeing fail our biological needs by not acknowledging that feelings of safety emerge from internal physiological states regulated by the autonomic nervous system. The study of feelings of safety has been an elusive construct that has historically been dependent upon subjectivity. Acknowledging that feelings of safety have a measurable underlying neurophysiological substrate would shift investigations of feelings of safety from a subjective to an objective science. Polyvagal Theory provides an innovative scientific perspective to study feelings of safety that incorporates an understanding of neuroanatomy and neurophysiology. This perspective identifies neural circuits that downregulate neural regulation of threat reactions and functionally neutralize defensive strategies via neural circuits communicating cues of safety that enable feelings of safety to support interpersonal accessibility and homeostatic functions. Basically, when humans feel safe, their nervous systems support the homeostatic functions of health, growth, and restoration, while they simultaneously become accessible to others without feeling or expressing threat and vulnerability. Feelings of safety reflect a core fundamental process that has enabled humans to survive through the opportunistic features of trusting social engagements that have co-regulatory capacities to mitigate metabolically costly defense reactions. Through the study of neural development and phylogeny, we can extract foundational principles and their underlying mechanisms through which the autonomic nervous system leads to feelings of safety and opportunities to co-regulate. Several principles highlight the validity of a science of safety that when implemented in societal institutions, ranging from healthcare to education, would enhance health, sociality, and lead to greater productivity, creativity, and a sense of wellbeing. By respecting our need to feel safe as a biological imperative linked to survival, we respect our phylogenetic heritage and elevate sociality as a neuromodulator that functionally provides the scientific validation for a societal focus on promoting opportunities to experience feelings of safety and co-regulation.

## Introduction

Contemporary strategies for health and wellbeing fail our biological needs by not acknowledging that feelings of safety emerge from inside the body. This paper focuses on feelings of safety, an elusive construct that has historically been dependent upon subjectivity. It is proposed that feelings of safety have a measurable underlying neurophysiological substrate. Acknowledging that feelings of safety are an emergent property of autonomic state would shift investigations of feelings of safety from a subjective to an objective science.

In writing this paper, I have reflected on a personal question – what principle can be extracted from conducting empirical research for more than five decades? What principle has captivated my passion and intellectual curiosity? What theme would I use to organize the information from my papers, books, and talks? Or, simply phrased what have I learned?

After reflecting on this question, I arrived at a concise and intuitive principle that humans, as social mammals, are on an enduring lifelong quest to feel safe. This quest appears to be embedded in our DNA and serves as a profound motivator throughout our life. The need to feel safe is functionally our body speaking through our autonomic nervous system - influencing our mental and physical health, social relationships, cognitive processes, behavioral repertoire, and serving as a neurophysiological substrate upon which societal institutions dependent on cooperation and trust function are based.

Feeling safe functions as a subjective index of a neural platform that supports both sociality and the homeostatic processes optimizing health, growth, and restoration. Operationally, feeling safe is our subjective interpretation of internal bodily feelings that are being conveyed via bi-directional neural pathways between our bodily organs and our brain. Feelings of safety are not equivalent to an objective measurement of safety, which may pragmatically be defined as the removal of threat. Feeling safe is more akin to a felt sense as describe by Gendlin (1997). Although Gendlin, as a philosopher and psychologist, was not physiologically oriented, he described a “felt sense” not as a mental experience, but as a physical one.

In understanding the motivation to feel safe, feelings of safety may to be conceptualized from the Polyvagal Theory. Polyvagal Theory provides an innovative scientific perspective that incorporates an understanding of phylogenetic shifts in vertebrate neuroanatomy and neurophysiology; this perspective identifies neural circuits that downregulate neural regulation of threat reactions and functionally neutralize defensive strategies via neural circuits communicating cues of safety. Feelings of safety are operationally the product of cues of safety, via neuroception (see below), downregulating autonomic states that support threat reactions and upregulating autonomic states that support interpersonal accessibility and homeostatic functions. Basically, when humans feel safe, their nervous systems support the homeostatic functions of health, growth, and restoration, while they simultaneously become accessible to others without feeling or expressing threat and vulnerability.

In explaining the profound importance of feeling safe, we are immersed in the ambiguity of our language when it comes to describing feelings and linking feelings to underlying neurophysiological states. This problem dates to the earliest psychologists such as Wundt (Wundt and Judd, 1902), who adopted and standardized introspection techniques to explore sensations, which we essentially limited to external stimuli. Polyvagal Theory leads toward a hierarchical conceptualization of feelings as higher brain interpretations of the neural signals conveying information regarding visceral organs (e.g., heart, gut, etc.) to the brainstem. This psychophysiological perspective emphasizes the foundational function of autonomic state in the subjective experiences of global feelings and specific emotions. Within this hierarchical conceptualization, feelings of safety are preeminent and form the core of an enduring motivational system that shifts autonomic state, which in turn drives behaviors, emotions, and thoughts. The resulting model suggests that feelings of safety reflect the foundational autonomic state supporting maturation, health, and sociality.

In an earlier paper (Porges, 1996), a hierarchical model of self-regulation was proposed to provide insights into optimizing intervention strategies for high-risk infants. The model reflects maturational competencies in neural regulation that provide a substrate for the more complex co-regulatory social behaviors. The main point of the model is that higher behavioral functions, which are frequently intentional, are dependent on the functioning of the more survival focused foundational systems embedded in the brainstem. The levels are described in Table 1. Level 1 is focused on the function of brainstem structures in optimizing physiological homeostasis through neural and neurochemical bidirectional communication between visceral organs and brainstem structures, which regulate the autonomic nervous system. The neural pathways involved in Level 1 are functional at birth in healthy full-term infants. An index of Level I can be derived from quantifying respiratory sinus arrhythmia, a periodic component of beat-to-beat heart rate variability that is synchronous with spontaneous breathing and a valid index of cardiac vagal tone via ventral vagal pathways (Lewis et al., 2012). However, in the preterm infant the system is not sufficiently mature, and the amplitude of respiratory sinus arrhythmia is notability low (Porges, 1992). Porges and Furman (2011) provide a more detailed description of the maturational changes in the neural regulation of the autonomic nervous system as a “neural platform” for social behavior.

**TABLE 1:** Hierarchical model of self-regulation (Porges, 1996).

Level I: Neurophysiological processes characterized by bidirectional communication between the brainstem and peripheral organs to maintain physiological homeostasis.
Level II: Physiological processes reflecting the input of higher nervous system influences on the brainstem regulation of homeostasis. These processes are associated with modulating metabolic output and energy resources to support adaptive responses to environmental demands.
Level Ill: Measurable and often observable motor processes including body movements and facial expressions. These processes can be evaluated in terms of quantity, quality, and appropriateness.
Level IV: Processes that reflect the coordination of motor behavior, emotional tone, and bodily state to successfully negotiate social interactions. Unlike those of Level III, these processes are contingent with prioritized cues and feedback from the external environment.

Level II emphasizes connections between higher brain structures and the brainstem in regulating autonomic state. Success in Level II is achieved when the suck-breathe-vocalize circuit is integrated with the ventral vagal pathway (Porges and Lipsitt, 1993). This circuit enables nursing and soothing to occur and is dependent on the neural pathways that define the ventral vagal complex (Porges, 1998). As higher brain structures, via corticobulbar pathways, regulate these brainstem nuclei of the ventral vagal complex, the pathways are subsequently repurposed as an integrated social engagement system, which foster social communication and co-regulation. It is through these connections that safety cues can recruit metabolically efficient states of calmness (e.g., slow heart rate) to optimize health, growth, and restoration. Or alternatively, threat cues can downregulate the social engagement system to optimize metabolically costly defensive strategies. The metabolic requirements for fight/flight behaviors require resources to be diverted from homeostatic functions. Autonomically this is observed through the disengagement of the vagal brake (Porges et al., 1996). In safe social settings the vagal brake is dynamically adjusting heart rate to match the metabolic needs of the behavior. The ability to disengage the vagal brake when motor behaviors, which are required in social interactions, are related to subsequent competencies in social behavior. More recently a new metric, vagal efficiency, was introduced to describe the dynamic “efficiency” of the vagal brake (i.e., cardioinhibitory pathways to the heart monitored by quantifying the amplitude of respiratory sinus arrhythmia) in regulating heart rate. This metric evaluates the slope of the regression line between short time periods (e.g., 15 s) of synchronous measures of heart rate and respiratory sinus arrhythmia. Functionally, the slope is providing an estimate of how much heart rate would change with a standardized unit change in the amplitude of respiratory sinus arrhythmia. This metric has been useful in evaluating sleep state in full-term newborns (Porges et al., 1999) and the maturational trajectory in pre-term infants (Porges et al., 2019). It is also sensitive to alcohol (Reed et al., 1999) and may serve as a potential indicator of dysautonomia, since it is greatly depressed in individuals with an adversity history (Dale et al., under review) and in those diagnosed with the hypermotility subtype of Ehlers-Danlos Syndrome (Kolacz et al., 2021). In the context of this chapter, Level II provides the foundational neural platform for feelings of safety and access to the circuits that would enable a neuroception of safety (see below).

Table 1 emphasizes the hierarchical nature of specific autonomic states and accessibility of behaviors that we cluster as self-regulation skills. The optimal function of each level is contingent on each of the preceding levels being adequately functioning. Observers of developing children are aware of the strong maturational influence that pushes the child through the sequence. However, few are aware of the parallels between development and evolution and how this information informs us regarding the adaptive functions of specific autonomic states. It is not that a specific autonomic state is good or bad, but rather what adaptive functions did ancestral vertebrates access while being in a specific autonomic state.

## Dissolution

Consistent with Polyvagal Theory (Porges, 2021a,b), the sequencing of the hierarchy of neural maturation mirrors features of vertebrate evolution. The theory emphasizes the modifications in the neural regulation of the autonomic nervous system that is highlighted through phylogenetic transitions, especially the transition from asocial reptiles to the sociality and co-regulation features of social mammals. Operationally defining feelings of safety as dependent on an autonomic state, provides an opportunity to study the potential emergent properties that are dependent on access to this state. Thus, it is proposed that the consequence of feeling safe provides the neural platform for cooperative behaviors, both supporting physiological systems and enabling accessibility to higher brain structures for learning, creativity, appreciation of aesthetics, and even spirituality.

An acknowledgment of this hierarchy, results in questions about the sequential unfolding of responses to challenges orienting within the body (e.g., fever and illness) and outside the body (e.g., threat). Disease and injury to the brain have been observed to disinhibit phylogenetically more ancient evolutionary structures, that in the healthy individual are regulated (e.g., inhibited) by newer brain structures. This was described by Jackson (1884), who stated that “the higher nervous arrangements inhibit (or control) the lower, and thus, when the higher are suddenly rendered functionless, the lower rise in activity.” Jackson labeled this process, dissolution, to emphasize that it is evolution in reverse.

While Jackson emphasized a dissolution process that mirrors the reverse of evolution in brain structures (i.e., moving from neocortex to lower brain structures), Polyvagal Theory emphasizes the reverse of evolution in the neural structures and pathways that regulate the mammalian autonomic nervous system. In this hierarchy of adaptive responses, the newest social engagement circuit is used first; if that circuit fails to provide safety, the older circuits are recruited sequentially. The elements of the social engagement system are functional at birth in the full-term infant (see Porges and Furman, 2011) and serve to enable infant and mother to co-regulate autonomic states via reciprocal cues of safety. The product of this co-regulation is the optimization of homeostatic functions enabling the infant to mature and the mother to recover from the metabolically demanding delivery process. Early in life this co-regulation provides the neurophysiological platform for mother-infant interactions and attachment (Bowlby, 1988), and the establishment of social bonds, which can be conceptualized as being dependent on associations with feelings of safety.

Focusing on Levels I and II we see that optimal behavior is dependent the neural regulation of the autonomic nervous system and the connectivity between cortical areas, allowing the accurate interpretation of cues of safety and threat, and the brainstem areas regulating the autonomic nervous system. The quantification of respiratory sinus arrhythmia provides a quantitative portal into Level I, while the vagal efficiency metric would reflect Level II competency.

## Autonomic State as an Intervening Variable

By placing autonomic state at the core of feelings of safety or threat, the pragmatic survival behaviors of fight and flight, as well as complex problem-solving strategies that would lead to escape, are consequential and dependent on the facilitatory function of the ANS in optimizing these strategies. Similarly, turning off threat reactions and calming autonomic state, via the ventral cardioinhibitory vagal pathway, will promote interpersonal accessibility, while simultaneously supporting the co-regulation of autonomic state. This model positions autonomic state as an intervening variable mediating the interpretation of contextual cues and shaping our reactions. Within this conceptualization, depending on the individual’s autonomic state, the same contextual cues and challenges may result in different behavioral, cognitive, and physiological reactions. For example, recent research documents that indices of autonomic state influenced the impact the pandemic on mental health (see Kolacz et al., 2020a), perceived stress in college students (Fanning et al., 2020), effectiveness of neurostimulation on abdominal pain (Kovacic et al., 2020), calming behavior in infants following the still face procedure (Kolacz et al., 2022), and protest behaviors in infants in daycare settings (Ahnert et al., 2021). This would be true both within and between individuals (see Porges et al., 2013). Thus, there may be a range of reactions among individuals who share the same environmental context, but who are in different autonomic states. In addition, the same individual may also have a range of reacting to repeated exposures to the same environmental context that would be mediated by variations in autonomic state. PTSD symptoms may be the product of a retuned autonomic nervous system following extreme and/or repeated exposures to threat. Research supports the conceptualization that the mental and physical health consequences of adversity are reflected in a retuned autonomic nervous system locked into states of defense that limited an access to the calming pathways through the ventral vagus associated with sociality (Williamson et al., 2013, 2015; Kolacz et al., 2020b).

Acknowledging the important role of autonomic state as an intervening variable would have profound consequences on our understanding of behavior and the often-faulty assumption that a behavior is intentional and reliably regulated by rewards and punishments. The model proposes that our cognitive intent and our bodily state can promote competing behavioral outcomes. As an observer of both behavior and autonomic state, my bet is on the potency of autonomic state. This conclusion is supported by the link between autonomic state and feelings of threat and our embedded biobehavioral program to survive. Since these states of defense are regulated by primitive neural circuits, circuits which are shared with many more ancient vertebrates, intentional self-regulation efforts originating in the cortex are frequently ineffective in downregulating survival driven reactions to threat, which are dependent on lower brain structures. The survival program is evolutionarily old, while the program that turns off threat reactions with cues of safety to promote calmness, sociality, and homeostatic functions is a mammalian innovation of a repurposed autonomic nervous system that may be influenced by higher brain structures. Although the calming system is effective in downregulating threat reactions in response to mild threats, it is difficult to access when the defensive systems are in a highly activated survival mode.

Functionally, we need to conceptualize the model as having both bottom-up and top-down pathways with the bottom-up pathways being a combination of being both reflexive and derivative of early evolutionary survival processes. The foundational are functionally hardwired via “neuroception” (see below). Thus, although cues of safety or threat will trigger a top-down reflexive changes in autonomic state, the states become associated with thoughts and behaviors. This process is initiated through interception and then bottom-up feelings of autonomic state are interpreted by higher brain structures, which in turn may initiate intentional behaviors. This linkage between feelings (i.e., autonomic state) and behaviors and thoughts form the neurophysiological basis for aspects of associative learning. The premise of many therapeutic strategies is to separate the feelings from the associative thoughts and behaviors. Strategies that are Polyvagal-informed focus on enabling the client to experience the feelings without linking the feelings to thoughts or behaviors (see Dana, 2018; Porges and Dana, 2018). Basically, the client learns that the feelings are not intentional or under voluntary control but are part of an adaptive reflexive system that is wired into our nervous system. Thus, although attributes of the sequence are initially reflexive, there are effective portals to modify the association. For example, acknowledging the hierarchy of organization of the autonomic nervous system would suggest that the introduction of cues of safety would be a functional antidote to threat reactions by reducing the associative links between feelings of threat and thoughts and actions. These speculations are consistent with dissolution, a process in which the cortical influence on regulating (i.e., calming) autonomic state become less effective. Functionally, the repurposed neural system that emerged during the transition from ancient reptiles to early mammals allowed sociality to function as a neuromodulator, calming physiology and optimizing bodily functions. In addition to sociality, positive memories and visualizations associated with positive experiences enable humans to access positive feelings (i.e., autonomic state) to actively inhibit threat reactions.

Access to sociality as a neuromodulator is influenced by both autonomic state and the flexibility or resilience that an individual’s autonomic state has in returning from a state of threat to a state that supports homeostasis. We also learn that this accessibility is, in part, dependent on a personal history during which autonomic state may have been retuned to optimize defense. This is frequently observed in individuals with a severe adversity history, whose traumatic experiences have retuned the autonomic nervous system to be locked in states of defense. This is reported by foster parents of children, who have been abused and for safety concerns have been removed from their biological parents.

## Culturally and Philosophical Influences Divert Interest in Feelings of Safety

Within our educational institutions we have been acculturalized to accept the dictum coined by philosopher Rene Descartes “I think, therefore I am” (cogito, ergo sum) (Descartes, 1986). This view has led to a cultural expectation that the rational mind defines us and that feelings distort this expectation and need to be constrained.

Polyvagal Theory provides an alternative perspective to this historical proposition. First, the theory would lead to a perspective that rather that thinking defining our existence, feeling does. Specifically, a revised Polyvagal-informed statement would suggest that I feel myself, therefore I am. I frequently use this example in my talks, although I use the French and not the Latin presentation of the dictum. Reflexive verbs are more commonly used in French than in English. Reflexive verbs are actions that the subject is performing upon itself. Thus, using the reflexive form of the verb to feel will unambiguously convey internal feelings and not the sensations of feeling (touching) an object. In English when we use the word feel, it is ambiguous and may reflect either situation. By using je pense, donc je suis, it is easily rephrased with the reflexive form of the verb to feel. The modified statement je me sens, donc je suis emphasizes that if I feel myself, I exist. A statement consistent with the current interest in embodiment and reports from trauma survivors of being disembodied and experiencing a bodily numbness.

Descartes’ philosophy led to a partitioning of human experience into separate domains dependent on mind (mental activities) and body (physical structure). Descartes proposed that mental states or processes cannot exist outside of the body and the body cannot think. The separation between mind and body, often labeled as Cartesian dualism, has been consistent with our contemporary cognitive-centric world view that is mirrored in a cortico-centric brain-body separation that dominates much of medical and mental health treatment models. Descartes argued that rationale decision making can only be developed when judgments are based not on passion (i.e., bodily feelings). This dualism is still prevalent in current medical practices, especially when illness cannot be linked to a specific organ dysfunction. When objective clinical assessments of bodily fluids and/or tissues do not provide a positive clinical indicator leading to an understood disorder, physicians often assume that the disorder is psychiatric or psychosomatic and the patient should get psychiatric consultation and care.

According to Damasio (1994), Descartes’ perspective had a fatal error in not acknowledging the interaction of feelings (i.e., body) with the mental activities (i.e., brain). Consistent with the Polyvagal Theory, Damasio (1994) emphasizes that bodily feelings can have a powerful influence on mental processes. Thus, rational thought, as espoused by Descartes would be a special case of mental processing in which the autonomic nervous system is not disrupting cognitive function. Perhaps, this special case is dependent on an autonomic state associated with feelings of safety.

Culturally, we have also been influenced by the concept of survival of the fittest. This concept was first introduced by Herbert Spencer (1851). Spencer proposed that individual self-preservation is the most important moral principle. The term was then used by Charles Darwin (1859) in On the Origin of Species. Darwin suggested that the organisms best adjusted to their environment were the most successful in surviving and reproducing. Over the decades “survival of the fittest” has frequently been interpreted as the strongest and most aggressive, which would suggest that control of resources and access to mating partners could be an objective metric of fitness and eventually the product of natural selection.

In the mid-20th century, a more integrated model emerged that began to reconcile the findings of genetics and inheritance with Darwin’s theory and its emphasis on natural selection. The resolution was called the evolutionary synthesis or modern synthesis, and one of its architects was Russian population geneticist Theodosius Dobzhansky. The key revelation was that mutation, by creating genetic diversity, supplied the raw material for natural selection to act on. Instead of mutation and natural selection being alternative explanations, they were joined in this new synthesis. This synthesis led to an alternative perspective of fitness. Dobzhansky’s insights lead to the following frequently quoted statement that “the fittest may also be the gentlest, because survival often requires mutual help and cooperation” (Dobzhansky, 1962). According to Dobzhansky, it is this capacity to cooperate that enabled the earliest mammalian species to survive in a hostile world dominated by physically larger and potentially aggressive reptiles.

Dobzhansky’s insightful statement converges on the emphasis of Polyvagal Theory on the phylogenetic transitions in neuroanatomy and neurophysiology as social mammals evolved from asocial reptiles. Mutual help and cooperation are dependent on a nervous system that has the capacity to downregulate threat reactions to allow the proximity necessary for cooperative behaviors and co-regulation. In mammals this is neuroanatomically and neurophysiologically observed in the repurposed neural circuits originating in brainstem areas that regulate the autonomic nervous system. The repurposed system enables feelings of safety to co-occur with sociality.

## The Phylogenetic Journey

Feelings of safety form the foundational neural platform for sociality. Through the lens of evolution, Polyvagal Theory focuses on how mammals adapted many of the phylogenetical ancestral structures that evolved to support survival in a hostile world. Note that the title of the initial publication presenting the theory (Porges, 1995) is a synopsis of the theory - Orienting in a defensive world: Mammalian modifications of our evolutionary heritage. A Polyvagal Theory. The title summarizes a phylogenetic narrative in which the survival of mammals was dependent on an ability to downregulate and modify the innate defensive systems that were inherited from their reptilian ancestors. These embedded vestigial circuits with their emergent adaptive functions are embedded in the genes of mammals. For mammals, whose survival is dependent on their sociality to cooperate, to connect, and to co-regulate (Dobzhansky, 1962), the ancient defense programs had to be harnessed and repurposed to enable the expression of signals of safety and calmness in proximity to another trusted mammal.

Polyvagal Theory’s interest in investigating mammalian autonomic regulation from a phylogenetic perspective does not focus on the obvious similarities with more ancient vertebrates. Rather, it focuses on the unique modifications that enabled mammals to optimize their survival. Consistent with this theme, Polyvagal Theory focuses on the evolved neural circuits that enabled mammals to downregulate the sympathetic activation that could support mobilization to fight or flee, to reduce psychological and physical distance with conspecifics, and to functionally co-regulate physiological and behavioral state.

The theory focuses on the transition from reptiles to mammals and emphasizes the neural adaptations that enable cues of safety to downregulate states of defense. Within Polyvagal Theory the evolutionary trend has led to a conceptualization of an emergent and uniquely mammalian social engagement system in which a modified branch of the vagus is integral. Neuroanatomically, this system is dependent on a brainstem area known as the ventral vagal complex. This area not only regulates the mammalian ventral cardio-inhibitory vagal pathway, but also regulates the special visceral efferent pathways controlling the striated muscles of the face and head. This does not preclude other structures from being involved in mammalian social engagement behaviors or homologous structures in other vertebrates who do not share our phylogenetic history being involved in social engagement behaviors.

The relationship between mothers and their nursing offspring illustrates the social engagement system in action. To survive mammalian offspring must initially nurse as their primary mode of ingesting food. To nurse the infant must suck, a process dependent on a brainstem circuit involving the ventral vagal complex. Survival is dependent on the infant’s nervous system efficiently and effectively coordinating suck-swallow-breathe-vocalize behaviors with vagal regulation of the heart through the ventral vagal pathways originating in the nucleus ambiguus. Through maturation and socialization, this “ingestive” circuit provides the structural neural platform for sociality and co-regulation, as major mediators, to optimize homeostatic functions leading to health, growth, and restoration (see Porges and Furman, 2011). For mammals there is a dependency between reactions to contextual cues and the functions of this circuit. Cues of threat may disrupt, while cues of safety may support or enhance functions. The sensory branches of the facial and trigeminal nerves provide major input into the ventral vagal complex. Functionally, changes in the state of this circuit, through the process of dissolution, will either disinhibit phylogenetically older autonomic circuits to support defense (e.g., predator, disease, physical injury, etc.) or inform all aspects of the autonomic nervous system, including the enteric system to optimize homeostatic function (Kolacz and Porges, 2018; Kolacz et al., 2019).

Mammals uniquely have detached middle ear bones, which distinguish them from reptiles in the fossil record. Detached middle ear bones delineate the frequency band that enables mammals to hear species-specific vocalizations associated with social communication and provide a “safe” frequency band in which they could communicate without detection by larger predatory reptiles. Middle ear bones are small bones that separate from the jawbone during gestational development and form an ossicle chain that connects the eardrum to the inner ear. Small muscles regulated by special visceral efferent pathways travelling through branches of the trigeminal and facial nerves regulate the transfer function of the middle ear and determine the acoustic properties of the sounds transduced through middle ear structures by controlling the stiffness of the ossicle chain. When the chain is stiff, the eardrum is tighter and low frequency sounds are attenuated; when the muscles relax, lower frequency sounds pass through the middle ear into the inner ear. In all mammalian species, based on the physics of their middle ear structures, there is a frequency band of perceptual advantage that is expressed when the middle ear muscles contract (see Kolacz et al., 2018). It is within this frequency band that social communication occurs, while the low frequencies that through evolution have been associated with predators are attenuated (see Porges and Lewis, 2010).

Interestingly, the coordination of the contraction and relaxation of these small muscles is frequently co-regulated with autonomic state and the muscles contract when there is strong ventral vagal tone to promote social communication and co-regulation. This coordination between listening to specific sounds and autonomic regulation, provides the neurophysiological basis for sound to communicate cues of safety and trust. In contrast, when the autonomic nervous system shifts to a state of defense the muscles relax, allowing detection of low frequency predator sounds, which support defense strategies with auditory cues. In this state, acoustic perception is biased toward detecting cues of threat.

The link between behavioral and autonomic state and listening is obvious in the study of language delays and auditory processing problems in children. Often children with problems in auditory processing also have behavioral state regulation limitations. This neurophysiological link identifies a potential portal to regulate autonomic state through acoustic stimulation, which is easily observable when a mother calms her infant using prosodic vocalizations. Similarly, we can observe this potent calming influence when a pet is calmed by the voice of a human. In addition, clinicians frequently report that survivors of trauma experience an auditory hypersensitivity to background sounds and an auditory hyposensitivity to human voices (Borg and Counter, 1989). These points further support the frequently observed link between hypersensitivity and poor autonomic regulation reflected in hyperarousal.

In a recent study (Kolacz et al., 2022), our research group documented that individual differences in maternal vocalizations had differential influences on calming infants, following an experimental manipulation known as the “still face” procedure (Tronick et al., 1978). Greater vocal prosody was more effective in calming behavior and reducing heart rate following the social disruption of the still face procedure. Of course, parents and pet owners are familiar with the impact of their voices on calming their children and mammalian pets.

Based on this link between prosodic vocalizations and calming, a listening intervention, known as the Safe and Sound Protocol™ (SSP), was developed to reduce auditory hypersensitivities, improve auditory processing, and calm the autonomic nervous system. The SSP functionally amplifies the embedded prosody in music by applying dynamic filters to pre-recorded music. Preliminary publications document the effectiveness of this strategy (Porges et al., 2013, 2014). The technology embedded in the SSP has received three patents and is marketed by Unyte/Integrated Listening Systems^1^. One of the awarded claims on the patents is for the application of the technology as an acoustic vagal nerve stimulator.

Through the evolution of vertebrates there were strong trends in the structures involved in regulating autonomic function. These trends may be summarized as moving from chemical to neural and then evolving greater specificity, efficiency, and speed through feedback circuits that relied on myelinated pathways. Evolution is a process of modification in which existing structures and circuits are modified to serve adaptive functions. In mammals, three primary autonomic states with specific neural circuits are observable and emerge at different times within the evolutionary history of vertebrates. In Polyvagal terms, the newest is labeled the *ventral vagal complex*, the oldest is the *dorsal vagal complex*, and in between is the spinal *sympathetic nervous system*. Thus, evolution informs us of the sequence through which the three phylogenetic dependent circuits regulate autonomic function in response to survival driven threat reactions. In humans, this sequence is replicated during gestation (see Porges and Furman, 2011).

## Neuroception

Polyvagal Theory proposes that the neural evaluation of risk and safety reflexively triggers shifts in autonomic state without requiring conscious awareness. Thus, the term “neuroception” was introduced to emphasize a neural process, distinct from perception, capable of distinguishing environmental and visceral features that are safe, dangerous, or life-threatening (Porges, 2003, 2004). A form of neuroception can be found in virtually all living organisms, regardless of the development of the nervous system. In fact, it could be argued that single-celled organisms and even plants have a primordial nervous system that respond to threat. As mammals, we are familiar with reactions to pain, a type of neuroception. We react to pain prior to our ability to identify the source of the stimulus or even of an awareness of the injury. Similarly, the detection of threat appears to be common across all vertebrate species. However, mammals have an expanded capacity for neuroception in which they not only react instantaneously to threat, but also respond instantaneously to cues of safety. It is this latter feature that enables mammals to downregulate defensive strategies to promote sociality by enabling psychological and physical proximity without an anticipation of potential injury. It is this calming mechanism that adaptively adjusts the central regulation of autonomic function to dampen the metabolically costly fight/flight reactions dependent on sympathetic activation and to protect the oxygen-dependent central nervous system, especially the cortex, from the metabolically conservative defensive reactions of the dorsal vagal complex (e.g., fainting, death feigning).

Polyvagal Theory proposes that neuroception functionally involves both top-down and bottom-up mechanisms. The process of neuroception, consistent with Level II in Table 1, is assumed to be initiated via top-down pathways involving cortical areas located in or near temporal cortex, components of the central nervous system that reflexively interpret cues of threat and safety. These areas of the cortex are sensitive to the intentionality of biological movements including voices, faces, gestures, and hand movements. Embedded in the construct of neuroception is the capacity of the nervous system to react to the intention of these movements. Neuroception functionally decodes and interprets the assumed goal of movements and sounds of inanimate and living objects. Thus, the neuroception of familiar individuals and individuals with appropriately prosodic voices and warm, expressive faces frequently translates into a positive social interaction, promoting a sense of safety. Autonomic state responds to the top-down detect of risk or safety. The autonomic reactions send sensory information regarding bodily feelings to the brain where they are interpreted and consciously felt. The bottom-up limb of the neuroception is functionally equivalent to interoception. Thus, although we are often unaware of the stimuli that trigger different neuroception responses, we are generally aware of our body’s reactions (i.e., visceral feelings) embodied in autonomic signatures that support adaptive behaviors (i.e., social engagement, fight/flight, shutdown).

## Social Connectedness: A Biological Imperative

A biological imperative identifies a need that must be fulfilled for a living organism to perpetuate existence and survival. Polyvagal Theory suggests that social connectedness is a core biological imperative for humans, since human survival is dependent on trusted others is wired into our genetics and is expressed throughout the lifespan starting from the moment of birth.

Polyvagal Theory proposes that social connectedness is tantamount to stating that our body feels safe in proximity with another. The theory elaborates that the neural structures involved in the Social Engagement System (Porges, 2009) orchestrate the autonomic states of the interacting dyad to both broadcast and receive cues of safety that downregulate threat reactions of defense and promote accessibility and co-regulation.

To be socially connected via a functional Social Engagement System (Porges, 2009), common brainstem structures must appropriately coordinate the striated muscles of the face and head with the vagal regulation of the viscera originating in a brainstem region known as nucleus ambiguus. Interestingly, neuroanatomically the special visceral efferent pathways regulating the striated muscles of the face and head originate and communicate with the brainstem area (i.e., ventral vagal complex) that regulate the ventral vagal cardioinhibitory pathway. The ventral vagal cardioinhibitory pathway provides the neural pathways that are expressed as the vagal brake and can be monitored by quantifying the amplitude of respiratory sinus arrhythmia.

An optimally resilient individual has opportunities to co-regulate physiological state with a safe and trusted other. Ideally, the “other” person projects positive cues regarding their autonomic state through prosodic voice, warm welcoming facial expressions, and gestures of accessibility. From an evolutionary perspective the integration of the neural regulation of the viscera with the regulations of the striated muscles of the face and head enable visceral state to be projected in vocalizations and facial expressions. This also allows vocalizations and facial expressions, modulated by autonomic states, to serve as cues of safety or threat to others. Together these pathways connect behavior to the nervous system and form the basis for social communication, cooperation, and connectedness. This system also produces, via a ventral vagal cardioinhibitory pathway, an autonomic state that produces feelings of safety and reflects an adaptive mastery of Level II processes (see Table 1).

Polyvagal Theory, by articulating an evolutionary hierarchy (i.e., based on Jacksonian dissolution) in the function of the autonomic nervous system to challenges, provides a guide to dynamically monitor adaptive autonomic responses. The autonomic state of an individual, serves as a functional map of the foundation for emergent behavioral, emotional, and physiological reactivity that an individual may have in response to threat or alternatively to positive experiences. The state of the autonomic nervous system provides a neural platform for an expanded range of feelings from threat to safety that provides a neurophysiological substrate for higher brain structures to elaborate these feelings. If the feelings are negative and dependent on autonomic states supporting defense, the feelings may evolve into diffuse states of anxiety or specific emotions such as fear or anger. Alternatively, if the feelings are positive and dependent on an autonomic state of calmness, thus enabling interpersonal accessibility and co-regulation, then these feelings may be associated with trust, love, and intimacy.

## Clinical Implications

From a Polyvagal perspective it may be helpful to investigate how challenges move us into physiological states of threat that would disrupt our connectedness and place our mental and physical health at risk. But, more relevant to both to clients and personal survival, therapists need to identify and emphasize the innate resources their clients have available to mitigate the potentially devasting reactions to threat, which in turn can destabilize the autonomic nervous system, sometimes resulting in visceral organ dysfunction and compromised mental health.

Awareness of the neural systems underlying Polyvagal Theory informs both therapists and clients regarding the threats to survival that can shift autonomic state, moving it through sequential neural platforms or states that mimic evolution in reverse or dissolution (Jackson, 1884). Functionally, to inhibit the trajectory of dissolution to calm, we must first use the competence of our social engagement system (a uniquely mammalian myelinated vagal pathway involving brainstem structures regulating vocal intonation and facial expressions) to connect with others and calm our physiology. Without these resources, we are vulnerable to move into adaptive defensive states.

Our defense repertoire is first expressed as chronic mobilization requiring activation of the sympathetic nervous system and then expressed as immobilization, which is controlled by an evolutionarily older unmyelinated vagal pathway. In the absence of an active social engagement system, the mobilized state provides an efficient neural platform for fight and flight behaviors. For many individuals this state will reflect chronic anxiety or irritability. When mobilization does not successfully move the individual into a safe context, then there is the possibility that the nervous system will shift into an immobilized state. Immobilization with fear can be associated with features of death feigning, syncope, dissociation, withdrawal, loss of purpose, social isolation, despair, and depression.

Although both defensive strategies have adaptive values in protecting the individual, they are dependent on different neural pathways (i.e., high sympathetic tone or high dorsal vagal tone). Activation of these systems, independently or simultaneously, will interfere with interpersonal interactions, co-regulation, accessibility, trust, and feeling safe with another person. Thus, defensive states emerge from neural platforms that evolved to defend, while simultaneously compromising capacities to downregulate our defenses through the coregulation with a safe and trusted individual. Basically, the theory emphasizes that in the presence of cues of safety, which we associate with positive social interactions, the mammalian social engagement system can downregulate our innate reactions to threat, whether the threat is tangible and observable or imagined and invisible.

## Stress and Threat Have a Common Neurophysiological Foundation

Several years ago, I wrote a paper on stress and tried to unravel the ambiguity of the term, especially the circularity of using stress as both a stimulus and as a response. Now after almost 30 years seeing the world through a Polyvagal perspective, as I write about safety and threat, I am reminded about my earlier approach to operationally define stress (Porges, 1985). It is easy to understand that the use of stress as a construct is ambiguous since stress has been operationalized to be a response as well as the contextual trigger producing the response. As this circularity is disentangled, note that the concept of threat has had a scientific history similar to stress in which removal stress is conceptualized as optimal and removal of threat is assumed to produce feelings of safety. Both constructs focus on the negative attribution of potentially observable and quantifiable features influencing biobehavioral processes, while originating outside of the body. Moreover, neither construct elaborates on the mechanisms that would optimize function following the removal of stress or threat, while implicitly assuming that removal is sufficient.

A Polyvagal perspective shifts the discussion from the external features defining stress and threat to the nervous system’s ability to support or disrupt homeostatic functions (i.e., processes supporting health, growth, and restoration). This new conceptualization would redefine stress as a measurable state during which homeostatic functions are disrupted. A redefinition would be consistent with the distinction between stress and coping by emphasizing that coping would functionally include an autonomic feature enabling a return to homeostatic function. It would also refine the dialog distinguishing between good and bad stress from the stimulus to the response. Thus, similar to threat, stress results in a retuned autonomic nervous system to support defense, while disrupting optimal bodily processes. If we assume that removal of stress and threat have the same autonomic signature in which autonomic state is disrupted and metabolic resources are diverted from homeostatic functions to survival needs. We could succinctly propose that feelings of safety would describe recovery to both stress and threat, since feelings of safety are dependent on a return to an autonomic state that would support homeostatic function. The ability to move, following a challenge, into an autonomic state that supports feelings of safety could also operationally define resilience.

Consistent with Cartesian dualism, there is an inherent acceptance that the external disruptor (i.e., stressor) can be operationalized and reliably result in a measurable “stress” response. In the 1985 paper I challenged the prevalent S-R model, which had been fundamental to experimental science, with an S-O-R model in which the O or organismic state measured by autonomic nervous system indices would function as an intervening variable mediating or moderating the influence of S on the R. Note that the S-R model is a behavioral restatement of the cause-and-effect relationship that has served as the basis of empirical science’s quest for laws of nature.

Because the intervening variable is assumed to be a source of response variability, researchers within the experimental laboratory-based disciplines are often uncomfortable and seek reassurance in comparatively simple interventions that are relatively independent of the individual differences associated with organismic variables. Among examples of this approach are manipulations of neural blockades (powerful drugs that block specific neural pathways), surgical severing of neural pathways, or brain lesions ablating specific areas of the brain. These manipulations are powerful and the impact on all participants is relatively similar. In the analyses of manipulation studies statistically significance is driven by the relative change to the manipulation versus the individual variations in response parameters among subjects. With powerful interventions strong relationships are easily observed and statistically confirmed. In contrast, using the S-O-R model, consistent with a Polyvagal perspective, we are looking for an interaction that will inform us about the features that distinguish between those who have strong or weak responses to the same manipulation.

In interpreting interactions with autonomic state (i.e., O) on the parameters of the R, there is often a discomfort within the experimental laboratory-based sciences. Thus, reporting interactions with state variables may provoke criticisms that the findings were due to a faulty hypothesis, poor experimental control, inappropriate study design, or a yet to be identified variable(s) influencing the gradation of reactivity. Frequently an alternative plausible explanation that the response (i.e., effect) is not deterministically related to the stimulus (i.e., cause) is not entertained. Fortunately, with the advent of robust multivariate statistical models, now there are accepted techniques that enable researchers to evaluate how other variables may indirectly, via intervening variables, mediate and moderate cause-and-effect relationships. These quantitative techniques were not commonly available during the early part of my research career. Below is the introductory segment of the 1985 paper.

*The construct of stress has a variety of definitions. Often the definitions appear circular, since stress has been defined in terms of environmental stimuli (i.e., a stressing environment), as an organismic vulnerability (i.e., a stress-prone organism), and as a response to the environment (i.e., a stress response). The inherent circularity of the definition has limited the succinct articulation of what stress is and what causes it.*

*Even if stress was operationally defined by labeling the stressing stimulus as the stressor and the behavioral and physiological response or adaptation to the stressor as stress, at least two problems would remain: (1) the definitions of stress and stressor would be circular, and (2) there would be situations in which individual and state variables mediate and modulate the degree of responsivity and adaptability (i.e., stress) of an organism to constant environmental manipulations (i.e., stressor). For example, the same environmental conditions that may result in physiological debilitation for one subject may not produce a discernible behavioral or physiological response in another subject or even in the same subject tested a second time. Thus, stress must be conceptualized not only in terms of the stressor and the observed response but also. In terms of the physiological state or vulnerability of the organism at the time of exposure to the stressor.*

*One approach to the complex problems associated with stress research would he to reformulate the research strategy. Research is generally conducted within the framework of a mechanistic stimulus–response (S-R) model. In this model the response variance is assumed to be determined by stimulus variance. Thus, stress responses would be determined primarily by the stressor. As noted above, however, the characteristics of the organism at the time of experimental manipulation would contribute to the manifestation of the stress response. Therefore, it would be expedient to use research designs that would enable the examination of the stress response not solely as the product of the stressor but also in terms of the state or condition of the organism prior to the stressor. The research would be formulated within the frame–work of a stimulus-organism-response (S-O-R) model. Thus, the changing characteristics of the organism might index vulnerability to the stressor and determine the degree to which the individual would experience stress.*

As we discuss stress, we note that experiences of stress and threat appear to reflect a common neurophysiological platform. Substituting threat for stress in the above paragraphs would result in similar conclusions. Stress, similar to threat, could objectively be defined as a disruption in homeostatic function. Basically, stress is triggering a bodily state of threat and reorganizing the autonomic nervous system to promote survival. Using this definition, we would be able to decouple good and bad stress (e.g., McEwen, 2013) as being defined by the durations and consequences of the disruptions. Short disruptions or acute stress followed by rapid recoveries would function as neural exercises promoting resilience. While more chronic disruptions without periods of recovery would lead to disease and tissue/organ damage. Research on perceived stress and coping (e.g., Folkman and Lazarus, 1984) that focuses on coping and the link between coping and positive emotions would be consistent with Polyvagal Theory’s emphasis on an operational definition of a stress response as prolonged disruption of homeostatic function and the powerful influence of cues of safety in downregulating threat.

A problem with using either threat or stress is that both constructs lead to binary (either/or) models in which removal of threat would be sufficient to feel safe and removal of stress would be sufficient to optimize homeostatic function and relaxation. Missing is an acknowledgment of the nervous system’s need for cues of safety and connectedness. These models could be reconceptualized to incorporate an understanding of safety and optimal homeostatic function, which might lead to operationally defined neurophysiologically based measures of recovery and resilience.

Others have proposed relationships between safety and stress (Bond et al., 2010; Dollard and McTernan, 2011; Brosschot et al., 2017; Slavich, 2020) that focus on constructs previously explored by the Polyvagal Theory (Porges, 2007). For example, the relationship between safety and stress forms the basis for the Generalized Unsafety Theory of Stress (GUTS) proposed by Brosschot et al. (2017). Although the two theories use different terms and constructs, the two theories can be contrasted if we assume that stress and threat responses are equivalent. The initial principle proposed in GUTS emphasizes that “the stress response is a default response.” In contrast, Polyvagal Theory proposes that stress is due to a disinhibition of a safety state and is not a default state. In fact, Polyvagal Theory would propose that stress (i.e., threat reactivity) rather than being a default state, can be reflected in two defensive states that would compromise homeostatic functions. The defensive states adaptively require foundational survival oriented autonomic states that differ from the safe state that supports homeostasis. To shift states, Polyvagal Theory proposes a process of dissolution, triggered by a neuroception of threat, that results in the disinhibition of evolutionarily older neural pathways that compromise homeostatic functions to serve foundational survival needs. By invoking the Jacksonian principle of dissolution, Polyvagal Theory proposes that stress is an adaptive product of the disinhibition of a safety circuit that supports homeostatic functions. This disinhibition will occur when survival is challenged by cues of danger, which trigger, via neuroception, the autonomic nervous system into states of defense. By acknowledging a hierarchy of autonomic states that parallel vertebrate evolution, the theory proposes a dissolution sequence in which more ancient autonomic circuits become available for defense. The critical points are: (1) stress is not a default state but a disinhibition of older survival circuits resulting in shifting the autonomic nervous system into states of defense, (2) autonomic state mediates the behavioral and physiological features of stress and safety, and (3) autonomic reactivity follows a predictable hierarchical sequence of disinhibiting evolutionarily newer circuits in the service of survival.

## Resilience: An Emergent Property of Connectedness and Feelings of Safety

Recently, I was interviewed for a documentary on resilience. The documentary was structured to define resilience through the personal narratives of three survivors of severe adversity. My role in the documentary was to provide a common theme capturing the essence of resilience from a Polyvagal perspective. The personal stories were emotionally penetrating and reflected very different experiences. One interviewee survived a suicide attempt by jumping off the Golden Gate Bridge. He related that at the moment he jumped, he knew he had made a mistake and knew he was loved and connected. He survived and has become an active suicide prevention speaker. Another interviewee was an award-winning chef with an incurable inflammatory disease that has impacted her eyes, skin, heart, liver, and kidneys. During the pandemic, she continued to pay her staff and used the remaining food in her restaurant to feed those in need. The third interviewee was a 19-year-old college student who contracted flesh eating bacteria. Twice during the initial treatments, his heart stopped, and he needed to be resuscitated. The consequence of the infection was that both legs and his fingers were amputated. Following his recovery he returned and graduated from college, climbed the highest mountain peak in Australia, plays golf, and started a foundation to help people like himself.

As I listened to their narratives, I felted humbled. How would I translate their courageous actions of survival and generosity into basic principles that were embedded in our biology? As I listened to their stories. two consistent principles emerged. First, they had a heightened degree of connectedness with others (e.g., family, community, and humanity). Second, this connectedness shifted them from a self-focused survival orientation to a concern for others with a sincere need to support others through actions of compassion, benevolence, and generosity. Even during their health challenges, they were signaling concern for the caregivers’ wellbeing.

Consistent with a Polyvagal perspective, their survival stories were narratives of individuals who had effectively fulfilled their biological imperative of connectedness. By intuitively connecting, they had tapped into a powerful resource, the social engagement system with its neurophysiological substrate, the ventral vagal complex. Even during the profound challenges of their illnesses, injuries, and personal losses, their nervous systems maintained a capacity to connect and to calm. This still leaves the important question of how some individuals efficiently recruit top-down mechanisms effectively accessing cues of safety from context and memories, while others do not. Polyvagal Theory would propose that this access is mediated by autonomic state and the efficiency of top-down mechanisms in regulating autonomic state. Potentially, this might be indexed by the vagal efficiency metric.

Polyvagal Theory informs us that sociality is a neuromodulator and functionally supports homeostatic functions that serve as a neurophysiological foundation for the positive emergent emotional and social consequences. In this paper, several related processes have been associated with feelings of safety or threat. In a sense, we could say that Polyvagal Theory emphasizes that resilience reflects a physiological state, which is sufficiently resilient to recover from disruptions, support feelings of safety, and connect with others via an active social engagement system. Moving into this state has positive outcomes that are not solely reflected in sociality and trust, but also in prosocial actions of compassion, benevolence, and generosity that enable humans to actualize their biological imperative of connectedness. The narratives highlighted the possibility that resiliency might be a product of a nervous system with sufficient resources to move out of the self-oriented focus of threat and stress to an other-oriented focus of feelings of safety that naturally emerge into actions of sociality, and compassion.

Resilience is a complex construct that appears to embody the successful integration of several skills and underlying neurophysiological mechanisms to recover from severe survival related challenges. Considering the focus and organization of this paper, resilience and feelings of safety share a common neurophysiological substrate. On its most foundational level, resilience reflects behavioral, physiological, emotional, and social processes that are dependent on the recovery of autonomic function to a state that supports social engagement as an adaptive strategy to co-regulate with others and to mutually support health, growth, and restoration. The antithesis of resilience might be thought of as being locked in an autonomic state that would support threat reactions within the body (e.g., injury and infection) and from others. In an autonomic state that supports threat, even the foundational processes described in Levels I and II in Table 1 would be compromised. When these foundational levels are functional, the nervous system can support coordinated goal directed behaviors. (Level III) and social interactions (Level IV).

An optimally regulated autonomic nervous system would support homeostasis and appropriately respond to challenges with an efficient vagal brake (i.e., enhanced vagal efficiency) by reacting and recovering to transitory challenges. But these narratives of resilience emphasize that there is a more integrative mechanism involved that is linked to fulfilling the biological imperative of social connectedness. This capacity for connectedness requires an active social engagement system, which broadcasts the individual’s accessibility through voice and facial expressivity. In a simplistic manner the face and voice, via brainstem pathways in the ventral vagal complex, provide a mechanism through which the autonomic state of two individuals can be shared and functionally transmit feelings of safety, trust, and accessibility that lead to an effective co-regulation. The ability to co-regulate is not simply a collection of voluntary operant behaviors involving facial expressions and vocalizations but seems to require the transmission of veridical cues of safety that are sufficient to elicit a neuroception of safety. It is hard to calibrate these cues, although we subjectively know through our personal reactions that with some, we feel safe and accessible, while with others we feel uncomfortable. I have come to label people, whose presence triggers a smile with feelings of accessibility and connection as super co-regulators. Perhaps, resilience, as a process, reflects the successful biobehavioral navigation of the four levels described in Table 1 and could succinctly be summarized as the capacity to spontaneously foster feelings of safety in both self and other.

## Concluding Remarks: Claiming Our Phylogenetic Heritage

Feelings of safety play a fundamental role enabling humans not only to survive, but to thrive. This supposition poses the important scientific question of how a feeling could be so critical to the survival of our species. To answer this question, it is first necessary to understand the relationship between feelings of safety and the specific neurophysiological architecture that underlies this specific category of feelings. Unfortunately, historical attempts to answer these types of questions have been elusive. Attempts to identify neurophysiological signatures of specific feelings, emotions, thoughts, or even global processes such as sociality have produced, at best, confusing and ambiguous results (e.g., Cacioppo et al., 2000; Levenson, 2003).

Central to the solution of this problem is how terms and constructs are used in different disciplines. In general, attempts to translate from psychological to physiological phenomena have focused on a strategy that could be succinctly labeled as “psychophysiological parallelism.” Psychophysiological parallelism is an intriguing strategy with a strong assumption that it is possible to identify unique neurophysiological signatures of specific mental processes (e.g., feelings, emotions, thoughts, etc.). In the 1960s psychophysiology emerged as an interdisciplinary science with historic roots embedded in this assumption.

Although Polyvagal Theory (Porges, 1995) emerged from traditional psychophysiology, it provided a theoretical demarcation from parallelism. In a sense, psychophysiological parallelism implicitly assumed that of the constructs employed in different domains were valid (e.g., subjective, observable, physiological) and focused on establishing correlations across domains that optimistically would lead to an objectively quantifiable physiological signature of the construct explored in the psychological domain. In contrast, Polyvagal Theory emphasizes the interactive and integrative aspect of different levels of the nervous system. The theory emphasizes a hierarchical organization that mirrors phylogenetic shifts among vertebrates. The evolutionary changes are also reflected in maturational trends. Thus, what appears to be more complex and related to higher brain structures, such as language and sensitivities to another’s physiological state via intonation of voice and gesture, is reflecting the functional and structural changes mapped into the evolutionary history of vertebrates. Frequently missed with our cortico-centric and cognitive-centric orientation is the importance of lower brain mechanisms in managing our basic survival-oriented reactions. Although the less complex earlier evolved systems are often repurposed in mammals, they remain survival oriented and are efficiently available to support states of defense when survival is challenged.

Psychophysiological parallelism is functionally a scientific strategy that assumes an isomorphic representation of a process with the gradations being mapped with equivalent precision on all levels. An alternate and more parsimonious strategy would be to organize the nervous system into a hierarchical format in which neurophysiological processes related to basic biologically determined survival needs are required to be managed successfully before higher brain structures are functionally given access to be activated for problem solving, creativity, and even sociality. Polyvagal Theory postulates that our biology is hierarchically organized with the basic survival needs, such as managing homeostatic functions, residing in foundational brainstem structures and optimal access and utilization of higher neural circuits being dependent on success at the foundational level. Polyvagal Theory provides insights into what the hierarchy is and how it can be identified and potentially monitored. According to the theory, the hierarchy reflects the phylogenetic shifts in the nervous system.

To reduce the intellectual burden of visualizing the evolutionary changes in the nervous system, Polyvagal Theory focused on the phylogenetic transitions of the autonomic nervous system. Specifically, the theory focused on the functional consequences of how the autonomic nervous system was repurposed during the transition from asocial reptiles to social mammals. This focus had a serendipitous benefit, since the brainstem area is relatively small and there was sufficient comparative neuroanatomy conducted to map the changes in the brainstem pathways and their potential adaptive functions. The theory focused on how phylogenetic changes in the neural regulation of the heart provided the foundational properties for feelings of safety and emergent sociality. This paper emphasizes that these two “psychological” constructs have a dependence on an evolved link between social engagement behaviors and vagal regulation. However, there are convergent evolutionary changes in how the neuropeptides of vasopressin and oxytocin function to support the advent of sociality in mammals (Porges, 2001c; Carter, 2021). Specifically, oxytocin plays important role in regulating the autonomic nervous system enabling mammals to immobilize without fear, give birth, and experience intimacy without recruiting physiological defensive reactions through dorsal vagal pathways (e.g., bradycardia, syncope, and diarrhea).

Information from three scientific strategies leads to an understanding of the critical role that feelings of safety play in human survival. Complementing the evolutionary and developmental trends introduced above there is a third strategy, an unfolding or de-evolution of the hierarchy in response to threat. This third strategy represents the study of pathology and adaptive reactions to challenges including threat and illness. Jackson (1884) formally introduced de-evolution as dissolution to emphasize the functional impact of disease or damage to the brain. He noted that as evolutionarily newer circuits became dysfunctional, they no longer inhibited the actions of older circuits, and the older circuits became active. Threat is a generalized response that can occur in response to a physical challenge, a pathogen, or even an inaccurate interpretation of context. The elements resulting a threat response do not have to be valid from an objective interpretation of threat. Rather, threat responses reflect the nervous systems interpretation of risk. In Polyvagal terminology, threat responses are due to a neuroception of danger or life threat in which the nervous system determines risk outside of conscious awareness. From a Polyvagal perspective, threat reactions represent a disruption of homeostatic function regardless of the validity of threat. Conversely, a neuroception of safety, results in an autonomic state that supports homeostatic function with emergent feelings of safety.

Feelings of safety and threat recruit different autonomic resources to optimize survival. However, survival as a construct is overly broad and confusing. Survival can initially be deconstructed into two domains of competence: one within the body and the other outside of the body. Although adequately responding to both challenges is necessary, internal competence needs to precede external competence. The internal processes focus on how the nervous system manages the regulation of bodily organs and supports the basic homeostatic processes of health, growth, and restoration. This is obvious when observing the challenge to survival of being born premature, basically born too soon for their nervous system to effectively manage homeostatic demands. The external processes focus on how the nervous system supports responses to challenges. For humans this starts with mastering the coordination of suck-swallow-breathe-vocalize behaviors that enable ingestion and social signaling.

On the most basic level, feelings of safety are a direct reflection of autonomic state when it is efficiently supporting homeostatic functions. But, there is a consequential factor. Being in a calm autonomic state provides neural access to the regulation of the muscles of the face and head that form an integrated social engagement system. This system utilizes the same neural network that coordinates sucking, swallowing, vocalizing, and breathing at birth. Interestingly, access to this system is also a portal to calm as evidenced by the overlap between social behavior and ingestion. Thus, feelings of safety not only reflect a calm autonomic state supporting homeostatic functions that are effectively regulated via the ventral vagus, but also provide access to the special visceral efferent pathways that also originate in the ventral vagal complex that enable social communication.

Frequently missed is the understanding that the brainstem area regulating the autonomic state that supports feelings of safety also regulates the muscles of the face and head that we use for ingestion, social communication, and signaling that we can be trusted and are safe to approach. For example, the intonation of voice reflects our autonomic state. If we are calm and our heart rate is slow and rhythmically variable, our voice is prosodic. The intonation of voice reflected in prosody is the product of vagal pathways regulating laryngeal and pharyngeal muscles. When we are frightened or angry our voice loses prosody, and our heart rate is fast and loses rhythmic variability. Feeling safe, by being in a calm autonomic state, provides access to efficiently use the social engagement system and to convey the feelings of safety to another. This is universally observed as a mother calms her infant with melodic vocalizations, gentle reassuring gestures, and warm facial expressions. Underlying the effectiveness of the mother’s vocalizations and facial expressions in calming the infant is the fact that these cues of safety are actively both reflecting the mother’s autonomic state and directly impacting on the nerves that regulate the infant’s autonomic nervous system via the same brainstem structures located in the ventral vagal complex. However, access to structures involved in ingestion and social engagement behaviors are limited by autonomic state. These structures are efficiently accessible when the autonomic nervous system is calm, not in a state of defense, and under the regulation of the ventral vagal pathway.

This paper has emphasized the common theme that feelings of safety reflect a core fundamental process that has enabled humans to survive through the opportunistic features of trusting social engagements that have co-regulatory capacities to mitigate the metabolically costly defense reactions. The short-term outcome is obvious in terms of the support of homeostatic functions. However, there is also a long-term consequence of feelings of safety that are reflected in the emergence of communities in which feelings of safety expand through spontaneous social engagement. Prosocial behaviors among a collective become the norm. Thus, our sociality enables an expansion of those with whom we feel safe and trust. This, of course, is the underlying premise of communities including legal systems, business transactions, political negotiations, and international treaties.

Through the study of neural development and phylogeny, we can extract foundational principles and their underlying mechanisms through which the autonomic nervous system leads to feelings of safety and opportunities to co-regulate. The study of mental and physical illnesses provides a convergent research strategy to confirm these principles, since illness is a trigger of dissolution, which functionally disrupts autonomic regulation and compromises social engagement behaviors.

In Table 2 above, the foundational principles are outlined. The principles succinctly form a hierarchy that leads to an optimization of health as well as mental, social, and behavioral processes. An acknowledgment of these principles in daily interactions and societal institutions would reinstate processes that would support the qualities of human experience, in which feelings of safety form the foundation of a healthier and more productive society. These principles highlight the validity of a science of safety that when implemented in societal institutions, ranging from healthcare to education, would enhance health, sociality, and lead to greater productivity, creativity, and a sense of wellbeing. In a way, by respecting our need to feel safe, we respect our phylogenetic heritage and elevate sociality as a neuromodulator and functionally provide the scientific validation for a societal focus on promoting opportunities to experience feelings of safety and co-regulation.

**TABLE 2:** Principles of a science of safety.

(1) Feelings of safety are a subjective interpretation of a calm autonomic state regulated by the ventral vagal pathway that supports homeostatic functions (i.e., health, growth, and restoration).
(2) Feelings of threat, stress, or anxiety are subjective interpretations of a shared defensive autonomic state that disrupts homeostatic function.
(3) Feelings of safety provide access to the social engagement system.
(4) When recruited, the social engagement system sends signals of safety (e.g., intonation of voice, facial expressions) to others that functionally downregulate (*via* neuroception) autonomic states of defense to states of calmness and accessibility mitigating the metabolically costly threat reactions through co-regulation.
(5) Co-regulation provides the neural state that supports the establishment of trusting relationships.
(6) Autonomic states of calmness (e.g., feelings of safety) enable efficient access to the higher brain structures involved in problem solving and creativity.
(7) The reciprocal benefits of co-regulation form the basis of sociality and support the neural systems optimizing health and performance.

In summary, feelings of safety and threat are subjective interpretations of the autonomic nervous system communicating via interoception with higher brain structures. As humans, we are on a life-long quest to feel safe. Polyvagal Theory deconstructs this intuitive truth into a plausible neuroscience with testable hypotheses and objective neurophysiological indices. Functionally, this quest to feel safe is the product of respecting the important functions of neuroception and the powerful role of co-regulation and other attributes of sociality as a neuromodulator that can optimize health, growth, and restoration.

## Author Contributions

SP contributed to the conceptualization and wrote the manuscript.

## Conflict of Interest

SP holds patent rights and receives royalties from UNYTE/Integrated Listening Systems for the Safe and Sound Protocol.

## Publisher’s Note

All claims expressed in this article are solely those of the authors and do not necessarily represent those of their affiliated organizations, or those of the publisher, the editors and the reviewers. Any product that may be evaluated in this article, or claim that may be made by its manufacturer, is not guaranteed or endorsed by the publisher.

## Footnotes

1
https://integratedlistening.com/porges/
